# Roles of Selected Bioactive Compounds in Inhibiting the Development and Progression of Cancer—A Review

**DOI:** 10.3390/ijms262110343

**Published:** 2025-10-23

**Authors:** Michaela Godyla-Jabłoński, Ewa Raczkowska

**Affiliations:** Department of Human Nutrition, Faculty of Biotechnology and Food Science, Wrocław University of Environmental and Life Sciences, Chełmońskiego 37, 51-630 Wrocław, Poland

**Keywords:** cancer, inflammation, oxidative stress, bioactive compounds, polyphenols, carotenoids

## Abstract

Natural bioactive compounds play an important role in regulating inflammatory processes and mechanisms of carcinogenesis. In view of the growing interest in their therapeutic properties, particularly in the treatment of chronic inflammation, cancer, and related diseases, this study reviews the molecular mechanisms of action of selected groups of compounds, namely polyphenols and carotenoids. The analysis is based on current scientific literature and evidence from in vitro and in vivo studies, with particular attention being paid to their effects on the NF-κB, STAT3, and MAPK regulatory pathways, as well as their role in modulating pro-inflammatory cytokine expression, apoptosis, and oxidative stress. These findings indicate that bioactive compounds represent a promising group of substances with a broad spectrum of biological activity. Nevertheless, their potential in combination therapy and in preventive strategies against cancer and inflammation requires further clinical investigation to clarify their bioavailability, safety, and therapeutic effectiveness.

## 1. Introduction

Cancer is one of the greatest public health challenges worldwide and remains the second most common cause of death. According to the latest GLOBOCAN 2022 estimates published by the International Agency for Research on Cancer, nearly 9.7 million deaths worldwide were attributed to cancer in 2022. By 2050, the number of new cancer cases is projected to rise to 35 million [1]. Cancerous diseases are characterised by the uncontrolled, rapid division of malignant cells that exceed natural growth limits. Unlike healthy cells, which strictly follow the body’s regulatory signals to maintain tissue homeostasis, cancer cells lose their ability to respond to such control mechanisms. This leads to unchecked proliferation, infiltration of neighbouring healthy tissues and organs, and, in advanced stages, the spread of the disease throughout the body via metastasis [2]. Inflammation, a natural defence mechanism of the body, can take two forms that influence cancer risk in different ways. Acute inflammation, typically caused by pathogenic attack, is short term and usually subsides once the threat is removed. Chronic inflammation, however, persists for extended periods and significantly increases susceptibility to cancer. At the core of most cancerous processes is the activation of two key inflammation-related signalling pathways: nuclear factor kappa B (NF-κB) and signal transducer and activator of transcription 3 (STAT3). Their excessive or prolonged activity drives uncontrolled cell growth and division, constituting one of the fundamental mechanisms of cancer progression [3]. Inflammatory diseases and cancer are thus closely connected. Chronic inflammation fosters cell transformation, proliferation, resistance to apoptosis, and metastasis [4].

Diet plays a crucial role in modulating the risk of developing many types of cancer. It can both promote and counteract carcinogenic processes. One of the clearest links is between obesity and an increased cancer risk. Excess body weight contributes to chronic inflammation, hormonal imbalances (e.g., elevated oestrogen and insulin levels), and metabolic changes, all of which create an environment favourable to the development of cancer cells. In addition, a high-fat diet—particularly one rich in saturated fatty acids (mainly trans fats)—can promote chronic inflammation. Such diets are often also low in dietary fibre, vitamins, and antioxidants, which are known for their protective properties. The absence of these nutrients weakens the body’s natural defences against DNA damage and cancer cell proliferation [5]. There is growing scientific evidence that bioactive substances of plant origin play a significant role in cancer prevention. These natural compounds, present in many foods, exhibit protective potential through a range of molecular mechanisms that influence carcinogenic processes [6,7,8]. In recent years, research on bioactive compounds such as polyphenols, carotenoids, flavonoids, triterpenes, and fatty acid metabolites has steadily increased, particularly regarding their role in modulating major pathways such as NF-κB, STAT3, phosphoinositide 3-kinase (PI3K/AKT)/mammalian target of rapamycin (mTOR), and mitogen-activated protein kinase (MAPK) [9,10]. Compounds such as curcumin, resveratrol, quercetin (QCT), and lycopene (LycT) are also widely studied for their anti-inflammatory and anticancer properties, as well as for their ability to enhance cancer cell sensitivity to chemotherapy [11].

At the same time, recent years have seen a significant increase in interest in hybrid strategies combining natural compounds with synthetic modifications, which improve pharmacokinetic properties, selectivity and therapeutic efficacy [12,13,14]. Dogra and Kumar (2023) emphasised that although many phytochemicals have documented anticancer activity, their limited bioavailability, stability and pharmacokinetic properties pose significant barriers to clinical application. The authors recommend the use of semi-synthetic analogues, metabolic engineering and delivery systems (nanotechnology) as crucial strategies for increasing the therapeutic potential of natural compounds [15]. In turn, Drabczyk et al. (2024) pointed out that biocompatible nanometric carriers (liposomes, polymer nanoparticles, liquid crystal nanostructures) enable precise drug delivery to the tumour, prolonged retention in cancerous tissues and a reduction in side effects for healthy tissues. As a result, even compounds with poor solubility can achieve significant therapeutic efficacy [16]. Furthermore, many recent studies shown the synergistic effects of drug combinations, for example, combining a natural compound with chemotherapy or signal pathway inhibitors, which allows for lower doses and reduced toxicity. Such strategies, combined with a precise delivery system, may represent the future of cancer therapy [17,18,19].

The aim of this study was to provide a comprehensive analysis of recent research on key bioactive compounds (berberine, curcumin, quercetin, resveratrol and lycopene) and their mechanisms of action in modulating inflammatory processes and carcinogenesis. Both in vitro and in vivo studies are discussed, along with clinical perspectives.

**Figure 1:**
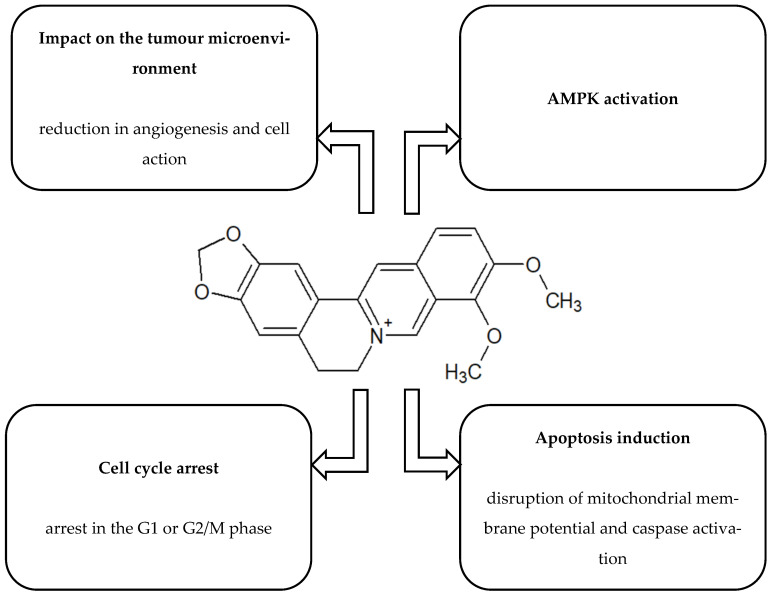
Structure and mechanism of action of berberine [20,21,22,23,24,25,26,27,28].

**Figure 2:**
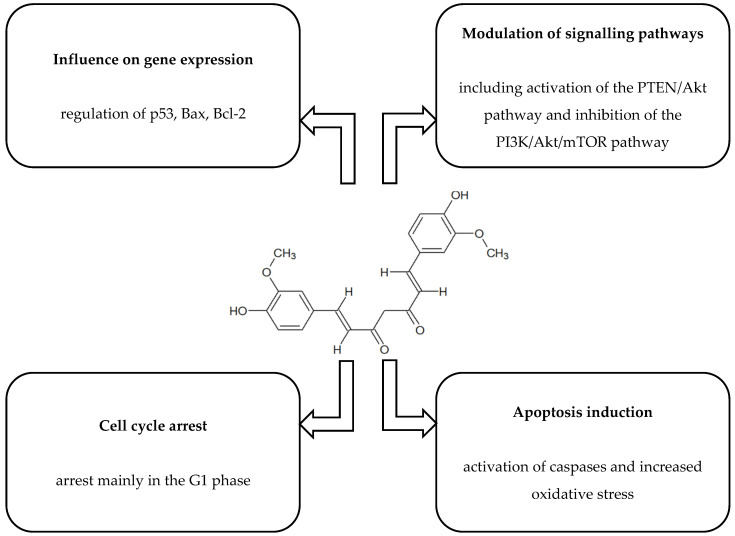
Structure and mechanism of action of curcumin [29,30,31,32,33,34,35].

**Figure 3:**
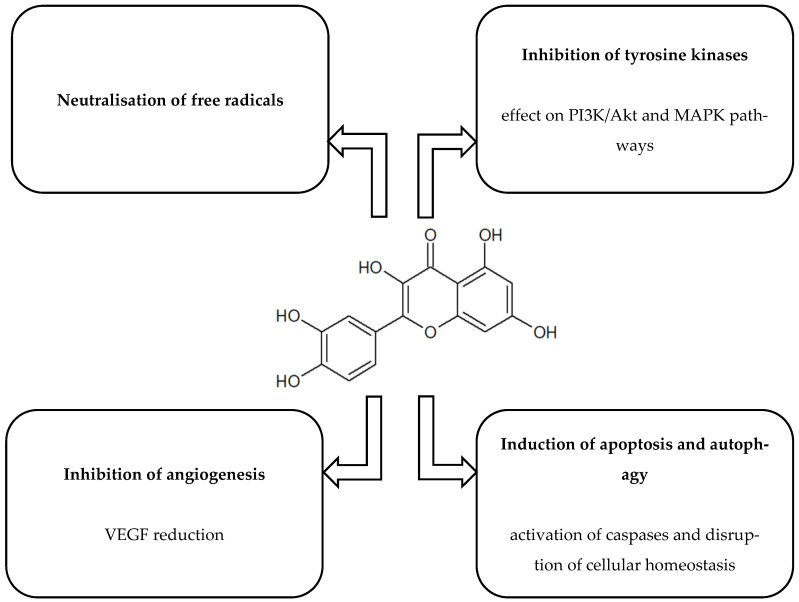
Structure and mechanism of action of quercetin [36,37,38,39,40,41].

**Figure 4:**
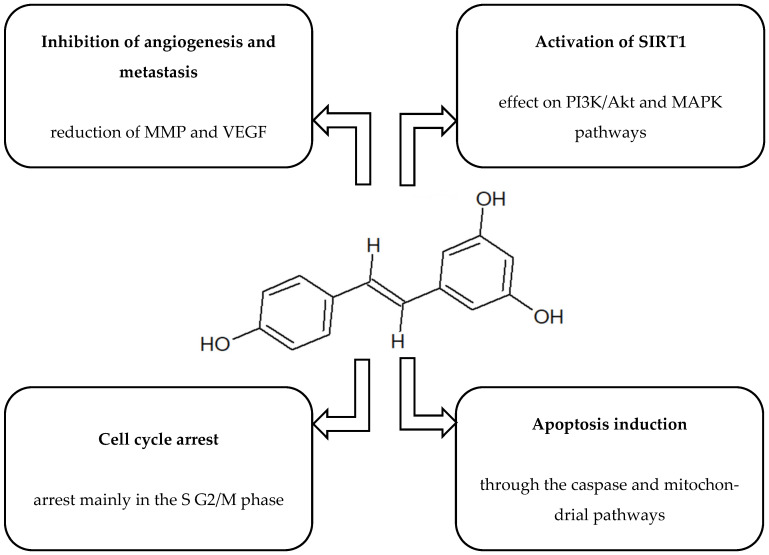
Structure and mechanism of action of resveratrol [42,43,44,45,46,47,48,49,50].

**Figure 5:**
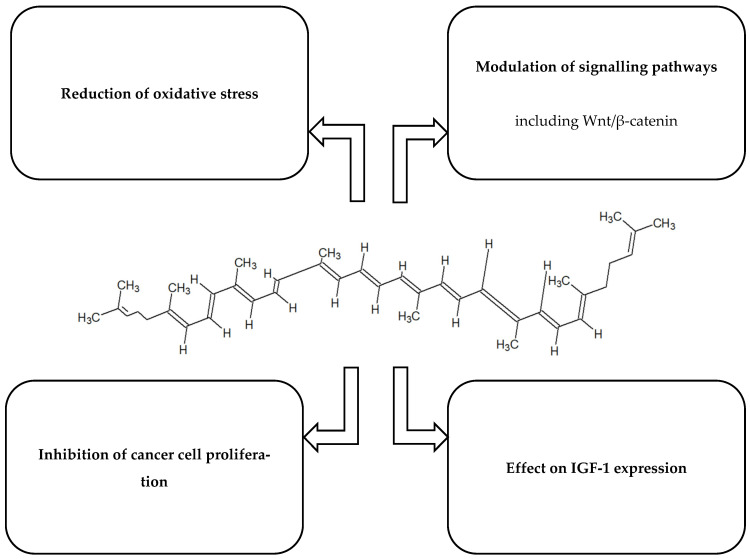
Structure and mechanism of action of lycopene [51,52,53,54,55,56,57,58].

Figure 1, Figure 2, Figure 3, Figure 4 and Figure 5 present the structures and mechanisms of action of selected bioactive compounds.

## 2. Effects of Selected Bioactive Compounds on Modulation of Tumour Proliferation and Inflammatory Processes

### 2.1. In Vitro Studies

Bioactive compounds of plant origin, such as polyphenols, alkaloids, and carotenoids, display strong anticancer and anti-inflammatory potential. Their mechanisms of action are multifaceted and include the inhibition of cell proliferation, induction of apoptosis, cell cycle arrest, and modulation of inflammatory signalling pathways such as NF-κB, AKT/mTOR, MAPK, and STAT3. Table 1 summarises selected in vitro studies that highlight the mechanisms of action of key bioactive compounds, with particular focus on berberine (BBR), curcumin, QCT, LycT, and resveratrol.

#### 2.1.1. Berberine (BBR)

BBR is an isoquinoline alkaloid isolated from plants of the *Berberis* genus, among others. It exhibits anticancer activity by inducing autophagy and apoptosis in hepatocellular carcinoma cells (*HepG2*, *MHCC97-L*, *SMMC-7721*) [26,77]. Another study reported its ability to inhibit the proliferation of *HepG2*, *Hep3B*, and *SNU-182* cells [59], as well as to reduce autophagy and proliferation in *MCF-7*, *4T1*, and *MDA-MB-231* breast cancer cells [27,78,79]. According to the findings of Yan et al. [62], BBR also shows promising anticancer activity against ovarian cancer, combining anti-inflammatory effects with reduced cancer cell proliferation and metastatic capacity. Its mechanism of action has been linked to the LINC00123/P65/MAPK10 signalling pathway, resulting in the modulation of glycolysis and autophagy in cancer cells. This discovery not only strengthens the potential of BBR as an adjunct to cancer therapy but also paves the way for the development of new therapeutic strategies. The results suggest that BBR may slow tumour growth by disrupting the energy processes critical for progression. However, the authors emphasise that the precise manner in which this molecular pathway regulates glycolysis and autophagy remains unclear and requires further investigation. Additional research, both at the cellular and molecular levels, is therefore needed to fully elucidate the mechanism of BBR in ovarian cancer [62]. In order to increase the therapeutic efficacy of BBR, it was encapsulated in liquid crystal nanostructures. This form showed strong anti-tumour properties against human lung adenocarcinoma cells (*A549*) by inhibiting the β-catenin pathway. At concentrations as low as 5 µM, a significant reduction in β-catenin expression was observed at both the mRNA and protein levels, which was further confirmed by simulation studies. Nanotechnology significantly improved the physicochemical parameters of BBR, including its bioavailability, stability and ability to target cancer cells, which translated into increased therapeutic efficacy [64]. This approach, typical for natural compounds with limited solubility and bioavailability, is finding increasingly wider application in oncology, enabling the overcoming of pharmacokinetic barriers and improving the selectivity of therapeutic action [80]. BBR binding at Ser663 further suggests the potential to block β-catenin phosphorylation. Nevertheless, this innovative approach requires further validation in preclinical studies [64]. A study by Xu et al. (2022) [28] revealed a strong cytotoxic effect of BBR on gastric cancer (GC) cells, independent of dose or exposure time. In vitro, BBR inhibited the proliferation of MKN-45 and HGC-27 cells, induced apoptosis, and arrested the cell cycle at the G0/G1 phase. Furthermore, it limited the migratory and invasive capacity of cancer cells, underscoring its therapeutic potential in GC treatment [28]. In order to elucidate the mechanism of action of berberine (BBR) in BGC-823 gastric cancer cells, experiments were conducted using inhibitors of the Akt, MAPK and mTOR pathways. Preliminary inhibition of pro-survival kinases enhanced BBR-induced autophagy, as confirmed by an increase in the number of MDC-positive cells. BBR inhibited the phosphorylation of Akt, ERK, JNK and p38 in a time-dependent manner, indicating its ability to induce cytostatic autophagy. Combination treatment with rapamycin enhanced cell death and reduced mTOR/p70S6K activity, suggesting that the mTOR pathway limits the cytotoxic effect of BBR. Additionally, the use of the autophagy inhibitor 3-MA partially reversed the changes in phosphorylation of the studied kinases, indicating the presence of a feedback mechanism in which autophagy enhances the anticancer effect of BBR by further suppressing pro-survival signals [61].

#### 2.1.2. Curcumin

Another compound is curcumin, derived from *Curcuma longa*, which shows strong antiproliferative activity in HCT116 colon cancer cells by inhibiting cyclin-dependent kinase 2 (CDK2 kinase), arresting the cell cycle in the G1 phase, and inducing apoptosis [35]. In pancreatic cancer cells (*BxPC-3*), inhibition of the epidermal growth factor (EGF)/extracellular signal-regulated kinase (ERK) and EGF/AKT pathways has also been observed [66], confirming its ability to affect growth receptors and tumour proliferation. Further research results indicate that curcumin exhibits cytotoxic effects on *MCF-7* breast cancer cells, reducing their viability in a dose- and exposure time-dependent manner. The mechanism of action involved the induction of apoptosis and increased caspase 3 and 9 activity. In addition, curcumin was observed to reduce miR-21 levels through activation of the PTEN/Akt pathway, which may be an important component of its anticancer activity [31]. A study conducted by Liu et al. (2018) showed that curcumin had a cytotoxic effect on human lung cancer cells (*A549*) in a manner dependent on both the duration of exposure and the dose used. The analysis also showed that this compound induced apoptosis in *A549* cells, with the intensity of this process increasing proportionally to the concentration of curcumin. Additionally, treatment with curcumin resulted in the activation of autophagy mechanisms, which was confirmed by the presence of fluorescent structures corresponding to autophagic vesicles (AV), an increased LC3-II/LC3-I ratio, and elevated Beclin1 levels, accompanied by a decrease in p62 protein expression. It was also observed that curcumin significantly inhibited the activity of the PI3K/Akt/mTOR signalling pathway. Importantly, pharmacological inhibition of mTOR (with rapamycin) or PI3K/Akt (with the inhibitor LY294002) potentiated the effect of curcumin, leading to increased apoptosis and autophagy and a marked reduction in cancer cell proliferation [32]. The use of combined therapy with curcumin and docetaxel (DTX) showed significant anticancer activity against oesophageal cancer cells, manifested by a reduction in their viability and migratory capacity (*p* < 0.01). At the same time, exposure to curcumin and DTX also led to increased apoptosis induction. Activation of the autophagy process was observed in cancer cells. Analyses suggest that this effect may be related to the inhibition of the PI3K/AKT/mTOR signalling pathway. The use of an autophagy inhibitor (3-methyladenine (3MA)) resulted in a reduction in autophagy induced by combination therapy, while simultaneously intensifying apoptosis and further limiting the proliferation of oesophageal cancer cells [33]. The use of curcumin in a colon cancer cell line (*LoVo*) model partially counteracted resistance to 5-fluorouracil (5-Fu) by reducing the ratios of phosphorylated forms of PI3K, AKT and mTOR relative to their total levels. Overexpression of MACC1 attenuated the effect of curcumin, leading to a rebound in the phosphorylation of PI3K/AKT/mTOR pathway proteins and a reduction in the effectiveness of curcumin in reducing resistance. Intervention with the BEZ235 inhibitor reversed the effect of MACC1 overexpression, restoring the sensitivity of cancer cells to 5-Fu. The results indicate that curcumin may reduce the resistance of colorectal cancer cells to 5-Fu by inhibiting epithelial–mesenchymal transition (EMT), which occurs through the regulation of the MACC1-dependent PI3K/AKT/mTOR pathway [34].

#### 2.1.3. Quercetin (QCT)

There are numerous studies in the literature confirming the beneficial effects of QCT on cancer processes. It is found in onions, apples, tea, and other substances. In vitro studies have shown that QCT exhibits anticancer properties against choriocarcinoma cells (*JAR*, *JEG3*), limiting their survival and proliferation. The mechanism of action involved increased production of reactive oxygen species (ROS), disturbances in mitochondrial potential, and cell cycle arrest in the sub-G1 phase. In addition, inhibition of PI3K/AKT pathway activity and activation of MAPK signalling were observed, leading to inhibition of cell progression. Combination therapy of QCT with chemotherapeutic agents (cisplatin, paclitaxel) showed a significantly stronger effect than each component alone, suggesting the potential of QCT as part of adjuvant therapy for gestational choriocarcinoma [36]. On the other hand, a pilot study by Zhu et al. (2017) [37] demonstrated that 7-O-geranylquercetin (GQ) can effectively induce apoptosis in GC cells (*SGC-7901* and *MGC-803*) by activating the ROS-dependent mitochondrial pathway and the MAPK cascade. Importantly, the cytotoxic effect of GQ on healthy gastric epithelial cells was negligible, which may indicate its selectivity. These findings suggest that GQ has therapeutic potential in GC treatment. However, to fully assess its candidacy for targeted therapy, further research is required into its mechanisms of action and effects on different cancer cell types [37].

Subsequent studies confirmed the ability of QCT to reduce the survival of Y79 retinoblastoma cells by arresting the cell cycle in the G1 phase. This effect was associated with decreased expression of CDK2/6 and cyclin D3, accompanied by increased levels of the inhibitors p21 and p27. In addition, induction of apoptosis involving caspase-3 and -9 was observed, along with disruption of the mitochondrial membrane potential (ΔΨm). Western blot analyses indicated activation of JNK and p38 MAPK following QCT treatment. Importantly, blocking these signalling pathways limited caspase activation and attenuated apoptosis [39]. To evaluate the effect of QCT on GC cells, in vitro experiments were conducted on *AGS* and *MKN45* cell lines. Analyses of cell proliferation and markers of ferroptosis and oxidative stress (iron, malondialdehyde [MDA], and ROS) confirmed a significant reduction in cancer cell viability and tumour size. A decrease in glutathione concentration and expression of key autophagic proteins (beclin 1, LC3B) was also observed. Genetic intervention using siATG5 reversed these effects, confirming the involvement of ferritinophagy, a process essential for the initiation of ferroptosis [81]. Experimental data have further shown that QCT can modulate oral cancer progression by regulating microRNA-16 (miR-16) and its molecular target, the HOXA10 protein. Treatment with QCT reduced viability, migration, and invasion of cancer cells, accompanied by lower matrix metalloproteinase (MMP)-2 and MMP-9 levels and reduced miR-16 expression. Overexpression of miR-16 confirmed its role in regulating cancer-related features, while restoration of HOXA10 weakened this effect, indicating a functional link between these molecules. Moreover, silencing miR-16 neutralised the effect of QCT, supporting its influence through the miR-16/HOXA10 axis [69]. Another study confirmed the potential of QCT as an anticancer agent against oral squamous cell carcinoma (OSCC) cells by examining its effects on cell viability, the cell cycle, and migration capacity [70]. In a cellular model of oral cancer, QCT demonstrated strong cytotoxic properties, reducing cancer cell viability without significantly affecting normal cells. Its mechanism involved ROS-mediated oxidative stress, cell cycle arrest in the S and G2/M phases, and activation of programmed cell death. At the same time, inhibition of migration and invasion was observed, accompanied by changes in the expression of proteins regulating apoptosis and extracellular matrix (ECM) degradation, including increased Bcl-2-associated X protein (Bax) and tissue inhibitor of metalloproteinases-1 (TIMP-1) levels and decreased Bcl-2, Bcl-XL, MMP-2, and MMP-9 levels [38]. These findings confirm the potential of QCT as a natural compound with anticancer activity, highlighting its possible role in the development of oral cancer therapies and supporting further evaluation of its efficacy in vivo and in clinical trials.

#### 2.1.4. Resveratrol

Resveratrol, like QCT, has been the subject of numerous studies evaluating its anticancer activity. Its natural sources include grapes and red wine. It influences many molecular mechanisms and displays multidirectional activity against oral cancer cells, including inhibition of proliferation, cell cycle arrest, and induction of programmed cell death. In addition, resveratrol reduces the migratory and invasive capacity of cancer cells and modulates their sensitivity to selected cytotoxic drugs. These mechanisms, although only partially understood, may significantly affect the course of carcinogenesis within the oral epithelium [82]. A study on resveratrol nanoparticles demonstrated their significant effect on oral cancer cells enriched with a population of cancer stem cells (CSCs). Treatment with resveratrol nanoparticles led to reduced cytokine levels, which correlated with decreased proliferation, invasion, and growth capacity of the CSC population. Furthermore, treatment lowered the expression of molecular markers associated with metastasis, such as prominin-1 (CD133), aldehyde dehydrogenase 1 (ALDH1), and C-X-C chemokine receptor type 4 (CXCR4) and angiogenic factors including MMPs, inducible nitric oxide synthase (iNOS), and vascular endothelial growth factor A (VEGF-A) [42]. An earlier study by Yu et al. [83] reported that resveratrol effectively inhibits OSCC cell proliferation, with the effect dependent on both treatment duration and concentration. In the analysed cell lines (*SCC-VII*, *SCC-25*, *YD-38*), IC_50_ values ranging from 0.5 to 1.0 μg/mL were observed after 48 h of treatment. Further analyses confirmed cell cycle arrest at the G2/M stage and increased expression of regulators of this stage, including phospho-cdc2 (Tyr15), cyclin A2, and cyclin B1. At the same time, a marked rise in apoptotic cells was observed, confirmed by flow cytometry analysis. These findings indicate that resveratrol may inhibit cancer cell growth through synergistic pro-apoptotic action and cell cycle regulation [83]. In turn, research by Chen et al. [45] showed that the therapeutic effect of resveratrol may be linked to blocking the activity of chromobox protein homologue 7 (CBX7) and attenuating signalling in the AKT pathway, while simultaneously activating mechanisms regulated by the p16 protein. This molecular regulation suggests that resveratrol acts by restoring tumour suppressor function and disrupting processes that promote cancer cell survival [45]. The process of tumour metastasis, including epithelial–mesenchymal transition (EMT), is a critical stage in disease progression. Research by Kim et al. [74] demonstrated that resveratrol can reduce the invasiveness and migration of OSCC cells by modulating the mitochondrial pathway and inhibiting transcription factors responsible for inducing EMT. These findings highlight the potential of resveratrol as a compound capable of interfering with mechanisms that promote metastasis [74]. Li et al. (2020) demonstrated that resveratrol exerts a cytotoxic effect on H446 small cell lung cancer cells, limiting their viability and inducing apoptosis. The mechanism of action involved increased cytochrome c expression, inhibition of the PI3K/Akt/c-Myc pathway, and translocation of AIF to the cell nucleus. Importantly, the use of NAC (an oxidative stress inhibitor) partially reversed these effects, indicating the involvement of reactive oxygen species and mitochondrial membrane potential disturbances in the induction of cell death. These results suggest that Res may activate programmed cell death in H446 SCLC cells through modulation of PI3K/Akt/c-Myc signalling and oxidative stress-dependent mechanisms [46]. A study by Liu et al. (2018) showed that resveratrol has significant anti-tumour effects on *A2780* and *SKOV3* ovarian cancer cells. This compound effectively reduced the proliferation, migration and invasion of cancer cells, while disrupting glycolysis and inducing apoptosis. Resveratrol increased AMPK and caspase 3 activity while decreasing mTOR kinase phosphorylation. Importantly, the use of an AMPK inhibitor (compound C) reversed the effect of AMPK and caspase 3 activation and restored mTOR activity, confirming the involvement of the AMPK/mTOR pathway in the mechanism of action of resveratrol [47].

#### 2.1.5. Lycopene (LycT)

LycT is a carotenoid found mainly in tomatoes and is known for its antioxidant and antiproliferative properties. In breast cancer cells (*MCF-7*, *MDA-MB-231*), LycT has been shown to induce apoptosis by increasing caspase activity and DNA damage [75]. In models of colon adenocarcinoma (*HT-29*, *LS174T*), LycT significantly inhibited proliferation, which may result from cell cycle arrest or reduced growth factor activity [52]. Particularly noteworthy findings were obtained in studies on SW480 colon cancer cells stimulated with lipopolysaccharide, where LycT markedly reduced the expression of inflammatory genes, including tumour necrosis factor α (TNF-α), interleukin (IL)-1β, IL-6, cyclooxygenase-2 (COX-2), and inducible nitric oxide synthase (iNOS). It also limited the production of nitric oxide (NO) and prostaglandin E2 (PGE_2_). This mechanism was associated with inhibition of NF-κB and JNK and p38 kinase activity, confirming the anti-inflammatory properties of LycT [76]. In a pancreatic cancer cell model, LycT was demonstrated to reduce the viability of *PANC-1* cells by inducing apoptosis, primarily through its strong antioxidant activity. This effect was linked to reduced oxidative stress at both cellular and mitochondrial levels and inhibition of NF-κB activation, a key regulator of pro-inflammatory and pro-proliferative gene expression. The observed reduction in cellular inhibitor of apoptosis protein 1 (cIAP1), cellular inhibitor of apoptosis protein 2 (cIAP2), and survivin, target proteins of the NF-κB pathway, correlated with increased caspase-3 activity and a shift in the Bax/Bcl-2 ratio toward pro-apoptotic signalling. These results confirm that LycT can modulate pancreatic carcinogenesis by influencing redox homeostasis and NF-κB-dependent signalling, highlighting its potential as a nutraceutical in cancer prevention [51].

### 2.2. In Vivo Studies (Animal Models)

In vivo studies have demonstrated that numerous bioactive compounds significantly limit tumour growth and regulate the inflammatory response in experimental animals. Among the most frequently studied substances are BBR, curcumin, QCT, resveratrol, and LycT, tested in mouse, rat, and chicken models with tumours of various organs including the lungs, stomach, colon, liver, breast, cervix, and ovary. An overview of these studies is presented in Table 2.

#### 2.2.1. Berberine (BBR)

BBR has long attracted interest as a potential anticancer agent. Numerous preclinical studies have evaluated its effects on different cancer types, both for prevention and treatment. A meta-analysis of 26 animal studies showed that BBR significantly reduced tumour volume and mass (particularly in breast and lung cancer) and inhibited angiogenesis without affecting the animals’ body weight [20]. The mechanisms of action of BBR include induction of apoptosis and inhibition of proliferation, migration, and invasion of cancer cells through modulation of the AMP-activated protein kinase (AMPK), MAPK, and AKT pathways [21,22]. In a xenograft model of gastric adenocarcinoma (MKN-45), BBR was shown to effectively inhibit tumour growth. An increase in the number of apoptotic cells was observed, from 5.86 ± 1.62% to 26.86 ± 2.99%. Transcriptomic analysis revealed that one of the principal regulatory mechanisms was the interaction of cytokines with their receptors, with a clear involvement of IL-6. Reduction in IL-6 levels translated into decreased tumour activity, indicating the central role of the IL-6/Janus kinase 2 (JAK2)/STAT3 pathway in the mechanism of action of BBR [28].

The efficacy of BBR has also been confirmed in the treatment of lung cancer in several animal models. In the Lewis lung cancer mouse model, use of the BBR nanocomplex with fullerene C_60_ (C_60_-Ber) at low concentrations resulted in a marked reduction in tumour metastasis to the lungs, exceeding the effect observed with free BBR [84].

By contrast, in the BALB/c nude mouse model, the derivative 8-cetylberberine demonstrated greater anticancer activity than the standard form of BBR. Oral administration of 8-cetylberberine at a dose of 10 mg/kg significantly inhibited tumour growth, surpassing the effect achieved with a much higher dose of BBR [65]. Additionally, in a lung cancer xenograft model in C57BL/6 mice, intraperitoneal administration of BBR at doses of 100–400 mg/kg for 4 weeks led to significant tumour growth inhibition, with the strongest effect observed at the intermediate dose (200 mg/kg). BBR treatment prolonged animal survival without adversely affecting body weight. Histological analysis revealed increased tumour necrosis and reduced expression of forkhead box protein M1 (FOXM1), a transcription factor, further supporting the potential of BBR in limiting tumour progression [85].

BBR also has the ability to modulate multidrug resistance. In a model of doxorubicin (DOX)-resistant breast cancer (MCF-7/DOX Fluc), its use in combination with DOX increased drug uptake and retention in tumour tissues and within cancer cells. The mechanism of action involved inhibition of P-glycoprotein and multidrug resistance-associated protein 1 transporters, which mediate the efflux of cytostatic drugs from cells. This inhibition significantly enhanced the therapeutic efficacy of DOX in vivo [105].

With regard to tumours associated with chronic inflammation, BBR demonstrated significant antitumour activity in an Apc^Min/+^ mouse model treated with dextran sulphate sodium. Treatment with dextran sulphate sodium increased the number of colon tumours, reaching a value of 12.83 ± 0.73, while the use of berberine resulted in their reduction to 7.78 ± 0.83. The reduction in colon cancer development was linked to decreased levels of pro-inflammatory cytokines (IL-6, TNF-α) in macrophages and inhibition of the EGFR/ERK pathway, which regulates cancer cell proliferation. In vitro experiments confirmed that BBR reduced the proliferative properties of intestinal epithelium carrying the Apc mutation, while administration of IL-6 in vivo neutralised its therapeutic effect, underscoring the key role of the inflammatory environment in its mechanism of action [86].

Overall, BBR exhibits multidirectional anticancer activity in various animal models, including inhibition of tumour growth, induction of apoptosis, modulation of inflammatory pathways, and enhancement of combination therapy efficacy. The accumulated evidence confirms its potential as a promising component of therapeutic strategies for the treatment of diverse cancers.

#### 2.2.2. Curcumin

Curcumin has been the subject of intensive research for many years due to its broad spectrum of biological activity, including anticancer properties. However, its limited bioavailability and rapid metabolism present significant challenges for therapeutic applications. To overcome these limitations, various strategies have been developed to enhance curcumin’s effectiveness, including the use of nanocarriers and the synthesis of structural analogues. One promising approach to overcoming the limitations of curcumin, such as low bioavailability and metabolic instability, is the synthesis of structural analogues. Of particular interest is a group of compounds known as 3,5-bis(arylideno)-4-piperidones (BAPs), also known as monocarbonylic analogues of curcumin. These compounds are characterised by greater chemical stability, a better pharmacokinetic profile and often stronger anticancer activity than curcumin itself [106]. Numerous in vitro studies have shown that BAP analogues exhibit strong cytotoxic effects against various cancer cell lines, including *HCT116* (colon cancer), *A431* (skin cancer) and *MCF-7* (breast cancer), often with IC_50_ values below 1 µM [107,108]. For example, compounds such as N-methyl-3,5-bis(arylidinyl)-4-piperidones exhibited IC_50_ values of 0.2–2.3 µM against B16 and L1210 cell lines, surpassing the activity of natural curcumin derivatives [109]. In some cases, high selectivity of action was also observed–limited cytotoxicity towards non-cancerous cells, indicating their potential clinical application [107]. A review of curcumin analogues emphasised that 3,5-bis(arylideno)-4-piperidone compounds not only exhibit strong anticancer and proapoptotic properties, but can also act as potent antioxidants and inhibitors of NF-κB and STAT3 [106].

In one in vivo study using BALB/c mice with 4T1 breast tumours, treatment with a folic acid–curcumin–gold–polyvinylpyrrolidone nanoconjugate significantly reduced tumour growth compared with the control group. Curcumin administered alone did not produce a comparable effect, highlighting the importance of targeted nanocarriers in improving bioavailability and selectivity of action [88].

Another in vivo study assessed the effect of orally administered curcumin (100 mg/kg) in mice with implanted breast tumours. Curcumin therapy effectively inhibited tumour growth, and its combination with physical exercise produced a stronger anticancer effect than treatment alone. Histological analysis confirmed the synergistic action of both interventions [89].

In colon cancer models, curcumin and its synthetic analogues EF31 and UBS109 demonstrated strong anticancer activity. These compounds inhibited cancer cell proliferation, reduced tumour growth in vivo, and enhanced the efficacy of chemotherapeutic agents such as oxaliplatin and 5-fluorouracil (5-FU). The mechanism of action involved blocking NF-κB activity, which led to reduced expression of angiogenesis factors, including hypoxia-inducible factor 1α (HIF-1α), COX-2, VEGF, and STAT3. EF31 and UBS109 were distinguished by favourable pharmacokinetic profiles and high efficacy at lower doses than curcumin itself, without showing significant toxic effects in animal studies [91].

In addition, a study conducted by de la Parte (2021) showed that oral administration of curcumin at a dose of 200 mg/kg for 28 days effectively reduced the growth of transplanted liver tumours derived from colon cancer in rats. A more than fivefold reduction in tumour volume was observed without adverse effects on serum biochemical parameters, confirming the safety of curcumin in this model [92].

Collectively, preclinical data indicate that curcumin and its structural or technological modifications (nanocarriers and synthetic analogues) exhibit significant anticancer activity in various cancer models, including breast, colon, and liver cancer. The mechanisms of action include inhibition of proliferation, angiogenesis, and NF-κB activity, as well as synergistic effects with other therapies. These findings support the potential of curcumin as a component of modern therapeutic strategies for cancer treatment.

#### 2.2.3. Quercetin (QCT)

QCT has also been the subject of numerous studies and is of interest as a compound with antioxidant, anti-inflammatory, and anticancer properties. In recent years, several preclinical studies have demonstrated its ability to modulate cellular pathways associated with apoptosis, proliferation, angiogenesis, and immune responses. A study by Roy et al. (2018) reported that a vanadium complex with QCT significantly reduced hyperplastic changes in female Sprague–Dawley rats, increasing the apoptosis index and decreasing the expression of pro-apoptotic markers (Bax, p53), while elevating the level of Bcl-2. A reduction in tumour cell proliferation was also observed (11.3 ± 0.12; 11.8 ± 0.10) [29]. Luo et al. (2024) further demonstrated that QCT alleviated depressive symptoms in mice exposed to chronic stress while simultaneously limiting breast tumour development under stressful conditions. The mechanism of action involved decreased STAT1 expression in microglia and reduced sympathetic activity within the tumour. The use of a colony-stimulating factor 1 receptor inhibitor confirmed the key role of microglia in this process [94].

In the transgenic adenocarcinoma of the mouse prostate (TRAMP) model, a diet enriched with QCT and resveratrol inhibited tumour progression by enhancing apoptosis and reducing oxidative stress. The combination therapy affected the expression of genes related to the cell cycle, methylation, androgen response, and the PI3K/AKT and phosphatase and tensin homologue (PTEN) pathways [95]. Another study showed that a ruthenium–QCT complex inhibited colon carcinogenesis in rats by increasing the levels of antioxidant enzymes, superoxide dismutase (SOD), catalase (CAT), and glutathione (GSH) (*p * < 0.01),and the expression of pro-apoptotic proteins (p53, Bax), while reducing Bcl-2 (*p * < 0.01). This effect was associated with increased apoptosis and reduced proliferation [40]. In a chronic lymphocytic leukaemia (CLL) model, QCT was shown to restore the sensitivity of radio-resistant B-1 cells to apoptosis by lowering Bcl-2 expression, normalising miR15a/16 levels, and limiting their migration to the liver [96]. In an acute myeloid leukaemia model, QCT exhibited epigenetic effects, reducing the levels of DNA methyltransferases (DNMTs), histone deacetylase 1 (HDAC1), and histone deacetylase 2 (HDAC2), while increasing the expression of pro-apoptotic genes, including death-associated protein kinase 1 (DAPK1), apoptotic peptidase activating factor 1 (APAF1), Bcl-2-like protein 11 (BCL2L11), and Bax. At the same time, QCT reduced STAT3/p-STAT3 activity [97]. The beneficial effects of QCT, particularly anti-inflammatory and anti-angiogenic, have also been demonstrated in lymphoma models. QCT modulated the AKT pathway by reducing phosphorylation of AKT and 3-phosphoinositide-dependent protein kinase 1 (PDK1), as well as the activity of proteins associated with cell survival, including Bcl-2-associated death promoter (BAD), glycogen synthase kinase 3β (GSK-3β), mTOR, and inhibitor of nuclear factor kappa-B α (IκBα). Concurrently, QCT increased PTEN levels and reduced expression of VEGF-A, COX-2, and iNOS [41].

The collected in vivo studies clearly indicate that QCT, both in its free form and in complexes with metals or as part of combination therapy, exhibits multidirectional anticancer activity. The mechanisms include induction of apoptosis, epigenetic modulation, reduction of oxidative stress and inflammation, modulation of the tumour microenvironment, and inhibition of proliferation and angiogenesis. These findings confirm the potential of QCT as a promising component of adjuvant therapy in the treatment of various types of cancer.

#### 2.2.4. Lycopene (LycT)

The natural carotenoid LycT displays a wide range of biological properties, including antioxidant and anti-inflammatory effects, and has therefore been the subject of numerous preclinical studies. In a model of N-nitrosodiethylamine (NDEA)-induced hepatocellular carcinoma, LycT supplementation effectively reduced tumour cell proliferation and metabolic disorders in liver tissue. Treatment stabilised the expression of genes related to the cell cycle (proliferating cell nuclear antigen, cyclin D1, and p21) reduced the activity of glycolytic enzymes and HIF-1α, and increased the number of apoptotic cells and macrophages, indicating a multidirectional mechanism of action [98].

A study by Bhatia et al. (2018) [99] confirmed the protective effect of LycT in the early stages of NDEA-induced HCC. Supplementation improved liver function, normalised haematological parameters, reduced pro-inflammatory cytokines (TNF-α, IL-6, IL-1β) (*p* ≤ 0.001), and strengthened the antioxidant system, highlighting LycT’s ability to modulate oxidative stress and inflammation [99]. In ovarian cancer, LycT supplementation was shown to significantly reduce both the incidence and size of cancerous lesions, particularly serous and mucinous adenocarcinomas. The anticancer effect correlated with decreased MDA levels, reduced NF-κB (by 38% at a dose of 200 mg/kg and 64% for 400 mg/kg (*p* < 0.001), respectively) and STAT3 activity (by 48% and 68%, respectively (*p* < 0.05)) and increased expression of nuclear factor erythroid 2–related factor 2 (Nrf2) (by 44% and 74%, respectively (*p* < 0.01 and *p* < 0.001)) and haeme oxygenase 1 (by 30% and 53%, respectively (*p* < 0.01 and *p* < 0.001), confirming the involvement of oxidative and inflammatory pathways in the mechanism of action of LycT. Lycopene supplementation significantly reduced serum MDA levels in hens compared to the control group (2.26 ± 0.51 nmol/mL), with the effect being clearly dose-dependent: 1.65 ± 0.39 nmol/mL for 200 mg/kg and 0.90 ± 0.27 nmol/mL for 400 mg/kg (*p* < 0.001) [100].

Cui et al. (2020) demonstrated that a diet enriched with LycT effectively counteracted oesophageal carcinogenesis in F344 rats. The strongest effect was observed at a dose of 25 mg/kg, which was associated with increased antioxidant enzyme activity, elevated peroxisome proliferator-activated receptor γ (PPARγ) and caspase-3 expression, and decreased NF-κB and COX-2 levels [101]. The same study also investigated photocarcinogenesis and showed that LycT can modulate the proliferation and apoptosis of keratinocytes exposed to UVB radiation. This mechanism involved regulation of forkhead box O3 (FOXO3a) activity, reduced activity of CDK2 and CDK4, increased Bax expression, and fragmentation of poly(ADP-ribose) polymerase (PARP). Additionally, the action of LycT was dependent on inhibition of the mechanistic target of rapamycin complex 2 (mTORC2)/AKT pathway, indicating its potential in the chemoprevention of skin cancer [53]. Similarly, a study by Koul et al. (2020) assessing LycT-enriched tomato extract in a chemically induced skin cancer model in BALB/c mice found that oral supplementation with LycT (5 mg/kg bw) significantly reduced the number (which was 82 in the group receiving 7,12-Dimethylbenz(a)anthracene (DMBA) and 12-O-tetradecanoyl phorbol-13-acetate (TPA) and 25 in the LycT + DMBA/TPA group), volume (24–333 mm^3^ and 0.3–156 mm^3^, respectively), and incidence of epidermal tumours (o 23.1%). This effect correlated with decreased expression of proliferative markers and angiogenic genes, including VEGF and angiopoietin-2, as well as increased expression of connexins 32 and 43 (Cx32, Cx43), suggesting improved intercellular communication. In addition, modulation of ECM components and a reduction in epidermal morphometric parameters were observed. These findings indicate that LycT exerts multidirectional effects, inhibiting proliferation, angiogenesis, and tissue architecture disruption during skin carcinogenesis, thus confirming its potential as a chemopreventive agent [54].

The presented preclinical studies clearly indicate that LycT exhibits multidirectional chemopreventive effects in various types of cancer. Its mechanisms of action include modulation of oxidative stress, inflammatory response, cell proliferation, and induction of apoptosis, highlighting its potential as an adjunctive substance in supportive cancer therapy.

#### 2.2.5. Resveratrol

Resveratrol, a natural compound from the stilbene group, has also shown anticancer activity in various models, particularly oral and cervical cancer. In animal models, resveratrol has been found to reduce the risk of tumour formation and limit tumour growth [82]. A study by Zhao et al. (2019) demonstrated that administration of resveratrol (10 mg/kg/day) to mice with transplanted cervical cancer cells (*HeLa*) significantly reduced tumour weight, an effect associated with modulation of the phospholipid scramblase 1 (PLSCR1) pathway [103]. Subsequent studies confirmed that resveratrol inhibits the proliferation of *HeLa* and *SiHa* cells in a dose-dependent manner, limiting their migration and colonisation. The mechanism of action involved blocking phosphorylation of STAT3 at tyrosine 705 (Tyr705), which suppressed EMT and ECM degradation. Treatment with resveratrol decreased levels of N-cadherin, vimentin, MMP-3, and MMP-13, while increasing E-cadherin expression, indicating a reduction in invasive potential [43]. To improve resveratrol’s bioavailability, an inclusion complex with hydroxypropyl-β-cyclodextrin (RHSD) was developed. In a cervical cancer model, RHSD showed stronger anticancer activity than free resveratrol, both preventively and therapeutically. Treatment with RHSD and free resveratrol resulted in a significant reduction in tumour growth. Their average volumes were 591.61 ± 112.78 mm^3^ and 719.52 ± 90.58 mm^3^ (*p* < 0.05), compared to the control groups, which recorded values of 992.3 ± 73.16 mm^3^ and 1102 ± 129.37 mm^3^. RHSD effectively reduced the expression of human papillomavirus (HPV) early protein 6 (E6) (*p* < 0.01) and early protein 7 (E7) (*p* < 0.01) oncogenes and increased levels of p53 (*p* < 0.01) and retinoblastoma protein 1 (Rb1) (*p* < 0.001) tumour suppressors, leading to apoptosis via these pathways [48]. Similar results were obtained in another cervical cancer model, in which resveratrol significantly reduced tumour weight and volume, decreased HPV E6/E7 mRNA and protein levels, increased p53 expression, and inhibited Rb1 phosphorylation. Immunohistochemistry confirmed reduced expression of viral oncogenes, demonstrating resveratrol’s efficacy in blocking the E6/p53 and E7/Rb1 pathways [44]. Beyond cervical cancer, resveratrol also exhibited protective effects in a model of colon cancer induced by 1,2-dimethylhydrazine (DMH). Treatment with resveratrol resulted in a significant reduction in oxidative stress, especially lipid peroxidation. Compared to the control group receiving only DMH, a significant reduction in the level of compounds reacting with thiobarbituric acid in blood serum was observed in subsequent stages of the experiment. It also increased the activity of antioxidant enzymes - glutathione peroxidase (increased by over 50% in the fifth month) and glutathione reductase (twice as high in the seventh month). Histological analysis showed that resveratrol inhibited the progression of neoplastic changes; in animals receiving DMH, adenocarcinoma in situ developed, while in individuals additionally treated with resveratrol, inflammatory changes in the colonic mucosa predominated [104]. In a xenograft model using *KTC-1* cells, the effect of resveratrol (30 mg/kg), rapamycin (3 mg/kg) and their combination on the activity of signalling pathways associated with tumour cell proliferation was assessed. After ten days of intraperitoneal administration, resveratrol was found to significantly inhibit AKT activation (*p* < 0.05), while rapamycin enhanced it. Combination therapy led to a marked reduction in AKT phosphorylation compared to rapamycin therapy alone. A similar effect was observed for p70S6K, whose activity was significantly lower after combination therapy than after rapamycin monotherapy. These results indicate that the simultaneous administration of resveratrol and rapamycin can effectively modulate the signalling pathways associated with cancer cell survival, making this combination a potentially promising therapeutic strategy for the treatment of papillary thyroid carcinoma. However, further preclinical and clinical studies are necessary to fully evaluate the mechanism of action and safety of this therapy [110]. Taken together, these findings confirm that resveratrol exhibits multidirectional anticancer activity. Its mechanisms include inhibition of proliferation, invasion, and metastasis, activation of apoptosis, and protection against oxidative stress and DNA damage. These results highlight the potential of resveratrol as a natural compound with chemopreventive and therapeutic value in cancer treatment.

### 2.3. In Vivo Studies Involving Humans

Increasingly more studies are highlighting the important role of phytochemicals in the prevention and treatment of cancer. Compounds such as carotene, flavonoids, BBR, and curcumin exhibit antiproliferative, pro-apoptotic, and immunomodulatory effects, making them promising candidates for adjuvant therapy. While much research still focuses on preclinical models, a growing body of epidemiological and experimental evidence supports their effectiveness. Clinical studies involving patients with cancer have examined the effects of natural bioactive compounds such as curcumin, BBR, QCT, resveratrol, and LycT on various aspects of tumour development and inflammatory responses. Although the number of well-designed clinical trials remains limited, current data suggest that these substances have promising therapeutic and supportive potential. A case–control study conducted in a Chinese hospital assessed phytochemical intake among adults with glioma compared with those without a cancer diagnosis. The findings showed that higher consumption of carotene, flavonoids, soy isoflavones, anthocyanins, and resveratrol was associated with a reduced risk of glioma. Statistical analyses using weighted quantile sum regression and Bayesian kernel machine regression indicated a particularly strong influence of anthocyanins and carotene. Non-linear dose–response relationships were also observed, suggesting that the protective effect may depend on intake levels. The authors emphasised the potential role of phytochemicals in glioma prevention, while noting the need for further epidemiological studies in this area [111]. Table 3 summarises selected human studies that highlight the mechanisms of action of key bioactive compounds, with particular focus on curcumin, berberine (BBR), LycT, QCT, and resveratrol.

#### 2.3.1. Curcumin

The compound most frequently analysed in clinical trials is curcumin. Available results suggest that it may be a promising component for the treatment of precancerous oral mucosal lesions and as an adjunct in PCa therapy, particularly in the context of designing further randomised trials. Saghatelyan et al. (2020) evaluated the effect of intravenous curcumin in combination with paclitaxel in women with advanced breast cancer. The combination therapy demonstrated greater clinical efficacy and improved quality of life without compromising safety [112]. In another study involving patients with the same type of cancer, supplementation with a mixture of phenolic compounds (curcumin, red clover, flaxseed, resveratrol) increased metabolite concentrations in tumour tissues and activated the p53/p21 pathway, resulting in cell cycle arrest (*p* < 0.05) and apoptosis of *MCF-7* cells (*p* < 0.05) [30]. The chemopreventive effect of curcumin was also shown in a randomised clinical trial involving 223 patients with oral leukoplakia. Six months of supplementation led to a higher clinical response rate compared with placebo, although no significant histopathological changes (*p* < 0.71) were observed. In the group receiving curcumin, a clinical response was observed in 75 patients, representing 67.5% of participants, while in the placebo group, this percentage was 62 patients (*p* < 0.03) [113]. Curcumin has further been evaluated as an adjunct to gemcitabine therapy in patients with advanced pancreatic cancer. A curcuminoid–lecithin complex demonstrated a favourable safety profile, stable quality of life, and 61.4% disease control, with a median progression-free survival of 8.4 months [114]. In a phase II trial of patients with metastatic castration-resistant prostate cancer (PCa), combination therapy with docetaxel and curcumin was well tolerated and effective, leading in some cases to normalisation of prostate-specific antigen (PSA) levels (14% of participants) [115]. By contrast, findings for colorectal cancer are inconsistent. In a study of patients with familial adenomatous polyposis, 1 year of curcumin supplementation did not produce significant changes in the number (*p* < 0.58) or size of polyps (*p* < 0.76) [117]. However, in smokers with aberrant crypt foci (ACF) in the rectum, monthly supplementation with a higher curcumin dose reduced ACF numbers by 40%, correlating with higher plasma levels of active metabolites [116]. Overall, curcumin exhibits multidirectional anticancer activity, but its effectiveness depends on the cancer type, dose, form of administration, and duration of intervention. These findings underscore the need for further clinical research to optimise therapeutic strategies.

#### 2.3.2. Berberine (BBR)

Another compound is BBR. Ravera et al. (2020) evaluated the effect of BBR on CLL cells collected from the peripheral blood of patients with CLL. BBR was shown to reduce the expression of anti-apoptotic proteins, including myeloid cell leukaemia 1 and Bcl-XL; disrupt oxidative phosphorylation; and increase cell sensitivity to venetoclax, thereby supporting their elimination [118]. A similar effect was reported by Mohammadlou et al. (2021), who observed reduced Bcl-2 and receptor tyrosine kinase-like orphan receptor 1 (ROR1) levels, along with an increased Bax/Bcl-2 ratio, activating the mitochondrial apoptosis pathway. In addition, BBR decreased the expression of miR-21, which has oncogenic activity [119]. In a separate study, Liu et al. (2017) assessed the effect of BBR on ectopic endometrial stromal cells isolated from patients with adenomyosis. BBR inhibited proliferation in a dose- and time-dependent manner, induced cell cycle arrest at the G0/G1 phase, and promoted apoptosis. Furthermore, BBR reduced the expression of pro-inflammatory cytokines IL-8 and IL-6; growth factors transforming growth factor β, EGF, and VEGF; and MMP-2. These findings indicate BBR’s potential to limit the invasive growth of ectopic endometrial stromal cells. Collectively, the results suggest that BBR may exert a protective effect in inflammation-induced adenomyosis progression, providing a basis for developing new therapeutic strategies [120].

#### 2.3.3. Lycopene (LycT)

LycT, a carotenoid with strong antioxidant properties, may play an important role in the prevention of hormone-dependent cancers, including breast cancer. One study evaluated the relationship between serum concentrations of selected carotenoids and the risk of breast cancer. Higher levels of α-carotene, β-carotene, LycT, and lutein/zeaxanthin were associated with a lower risk of disease in both premenopausal and postmenopausal women. A stratified analysis according to oestrogen receptor and progesterone receptor status confirmed an inverse relationship between these compounds and breast cancer risk, regardless of receptor subtype. By contrast, β-cryptoxanthin showed no significant association with disease risk [126]. A study by Kim et al. (2018) demonstrated a significant inverse relationship between total carotenoid intake and the risk of GC (*p* for trend = 0.012), particularly among women (*p* for trend = 0.039). Higher LycT intake was associated with a lower risk of GC in the general population, as well as in men, women, *Helicobacter pylori*-infected individuals, and smokers. Among the main sources of LycT, tomato and tomato ketchup consumption was most strongly associated with reduced disease risk. These findings suggest that a LycT-rich diet may exert a protective effect against GC [127]. As part of a systematic review and meta-analysis, Rowles et al. [44] analysed the dose–response relationship between LycT intake (both dietary and blood concentrations) and PCa risk. Forty-two studies involving more than 690,000 participants and more than 43,000 cases of PCa were included. The results showed that higher LycT intake and higher blood concentrations were significantly associated with a reduced risk of disease (relative risk = 0.88). Dose–response analysis revealed that each additional 2 mg of dietary LycT was associated with a 1% reduction in risk, while each 10-μg/dL increase in blood LycT corresponded to an approximately 3.5% risk reduction. Although no significant effect on the risk of advanced PCa was observed, a trend toward protection against more aggressive forms was noted. The authors emphasised the need for further research into the biological mechanisms underlying these associations [128]. In a randomised clinical trial, Paur et al. (2017) evaluated the effect of a 3-week dietary intervention with tomato products (providing 30 mg of LycT per day), either as monotherapy or in combination with selenium, omega-3 fatty acids, and other bioactive components, on PSA levels in 79 patients with non-metastatic PCa. The main analysis showed no significant differences between the intervention and control groups. However, post hoc analyses revealed that PSA levels were significantly reduced in patients with intermediate disease risk and in those with the greatest increases in plasma LycT, selenium, and C20:5 n-3 fatty acid concentrations (*p* < 0.016). These findings suggest that the effectiveness of LycT interventions may depend on tumour aggressiveness and individual metabolic responses to dietary components [122].

Epigenetic studies conducted among survivors of head and neck cancer identified three distinct DNA methylation profiles, each characterised by a specific pattern of inflammatory pathway activity, including those related to cytokine signalling, B-cell receptors, and T-cell receptors. In one cluster, elevated serum LycT levels were observed alongside changes in the methylation of immune signalling genes, such as hypermethylation of CD40 ligand (CD40LG), alterations in Tec protein tyrosine kinase, and hypermethylation of T-cell surface glycoprotein CD8 alpha chain (CD8A). In another cluster, typical epigenetic regulation of the Toll-like receptor pathway was noted, combined with hypermethylation of mitochondrial ribosomal genes and higher alcohol consumption. The observed associations between methylation status, inflammatory biomarkers, alcohol intake, and LycT concentration suggest a possible influence of environmental and dietary factors on epigenetic regulation in patients with a history of head and neck cancer. These relationships warrant further investigation because they may provide a basis for future clinical recommendations and personalised prevention strategies [123].

#### 2.3.4. Quercetin (QCT) and Resveratrol

QCT and resveratrol are compounds less frequently investigated in human studies. QCT has been shown to regulate microRNA expression in patients with lung cancer, including an increase in suppressor microRNAs (let-7, miR-146a) and a decrease in the oncogenic microRNA miR-17, which may be relevant for controlling cancer progression at the epigenetic level [124]. Resveratrol, by contrast, has been tested in patients with colorectal cancer, where it reduced cell proliferation (demonstrated by decreased Ki-67 levels) and showed good penetration into intestinal tissues, confirming both its bioavailability and potential efficacy in chemoprevention [125].

Despite growing interest in naturally occurring bioactive compounds such as resveratrol, curcumin, BBR, LycT, and QCT, the number of clinical studies evaluating their impact on cancer development and progression in humans remains limited. Conclusive evidence of their clinical efficacy is still lacking, hindering the integration of these substances into standard therapeutic regimens. A further challenge lies in the low bioavailability of many such compounds, due to limited solubility, rapid metabolism, and instability in physiological environments. To address these limitations, intensive research is being conducted on modern delivery systems, including nanoparticles, liposomes, and inclusion complexes. Although preliminary results are encouraging, the efficacy and safety of these technologies require rigorous validation. At the same time, knowledge of the molecular mechanisms of action of these compounds continues to expand, although many aspects remain unclear. A thorough understanding of their interactions with cancer cells is crucial for optimising dosing strategies, predicting therapeutic responses, and identifying patient groups most likely to benefit. It is also worth noting that although these substances are widely regarded as safe, potential side effects and interactions with other drugs must be carefully evaluated. Taking these factors into account is essential for ensuring the effective and safe application of bioactive compounds in the treatment of patients with cancer.

This literature review stands out from previous studies due to its broad, interdisciplinary approach to the subject and the integration of data from in vitro, in vivo (including clinical) and epidemiological studies, with particular emphasis on natural bioactive compounds with anti-cancer and anti-inflammatory properties (berberine, curcumin, quercetin, resveratrol, lycopene). Unlike previous reviews, quantitative data (including IC_50_, percentage of proliferation inhibition, effective doses) have been collected and presented, allowing for a comparison of the potency and assessment of the therapeutic potential of individual compounds. In addition, the article covers nanostructures and delivery systems (e.g., liquid crystal nanostructures, liposomal carriers) that increase the bioavailability and selectivity of natural compounds. The review also includes data on synergy with chemotherapeutic drugs and the possibility of reducing doses and toxicity while maintaining anticancer efficacy. Particular emphasis is placed on dual action (anti-inflammatory and anticancer), which is crucial in the context of modern therapeutic strategies and cancer prevention.

## 3. Materials and Methods

This article reviews the scientific literature on the impact of selected bioactive compounds on inflammatory pathways and mechanisms associated with carcinogenesis. The literature search was carried out in the electronic databases PubMed, Scopus, Web of Science, and Google Scholar. Publications published in English between 2015 and 2025 were included. The search strategy used AND/OR operators with the following keywords: bioactive compounds, inflammation, cancer, carcinogenesis, inflammatory pathways, NF-κB, STAT3, polyphenolic compounds, berberine (BBR), curcumin, lycopene (LycT), resveratrol, anticancer mechanisms, and nutraceuticals. The analysis considered articles reporting in vitro and in vivo experimental studies on the effects of bioactive compounds on inflammatory mechanisms and cancer processes, as well as clinical studies evaluating supplementation with bioactive compounds in relation to inflammatory biomarkers and cancer risk. The structures of the chemical compounds discussed were developed using ChemSketch 2025.1.0.

## 4. Conclusions

Bioactive compounds of natural origin, such as BBR, curcumin, QCT, LycT, and resveratrol, show considerable potential in modulating inflammatory pathways and mechanisms involved in cancer development. Both in vitro and in vivo studies highlight their ability to inhibit pro-inflammatory cytokine activity; regulate key signalling pathways such as NF-κB, MAPK, and STAT3; induce apoptosis; inhibit proliferation and angiogenesis; reduce oxidative stress; and support cellular defence mechanisms. Despite these promising findings, evidence from clinical studies remains limited. The efficacy and safety of bioactive compounds in humans still require further verification through well-designed trials.

Bioactive compounds hold significant promise as adjuncts to conventional cancer therapies. However, further translational research and large-scale clinical studies are essential to determine optimal dosing strategies, improve bioavailability, evaluate potential drug interactions, and establish the long-term safety of their use.

## Figures and Tables

**Table 1:** A review of in vitro studies on the effect of selected bioactive compounds on cancer cell proliferation and inflammatory processes.

Reference Source	Compound	Study Group/Tumour Type	Impact on the Organism/Mechanism of Action
[26]	BBR	Human hepatic carcinoma cell lines (*HepG2* and *MHCC97-L*)	Induction of autophagy and apoptosis
[59]		Human hepatic carcinoma cell lines (*HepG2*, *Hep3B*, and *SNU-182*)	↓ Cell proliferation
[60]		Human hepatic carcinoma cell lines (*Huh7* and *HepG2*)	Enhancement of radiosensitivity (via suppression of the Nrf2 signalling pathway)
[27]		Human hepatic carcinoma cell lines (*SMMC-7721* and *HepG2*)	↓ Cell proliferation, induction of apoptosis
[61]		GC cell line (*BGC-823*)	↓ Cell proliferation, enhancement of autophagy
[28]		GC cell lines (*MKN-45* and *HGC-27*)	↓ Cell proliferation, induction of apoptosis, cell cycle arrest at the G0/G1 phase, ↓ migration and invasion of GC cells
[27]		Breast cancer cell line (*MCF-7*)	↓ Autophagy, ↓ cell proliferation
[62]		Human ovarian cancer cell lines (*SKOV3* and *HEY*)	↓ Cell proliferation
[63]		Human lung cancer cell lines (EGFRm NSCLC)	↓ Cell survival, ↓ cell proliferation
[64]		Adenocarcinoma human alveolar basal epithelial cell line (*A549*)	Downregulation of β-catenin gene and protein expression
[65]		Human non-small lung cancer cell line (*A549*)	Induction of cell cycle arrest at the G1 phase (disruption of cyclin D1 and E1 expression), ↓ apoptosis (induction of the caspase pathway)
[23]		Colorectal cancer cell line (*HCT 116*)	Cell cycle arrest at the G0/G1 phase, ↓ telomerase activity in cancer cells, ↓ concentration of TERT and TERC
[24]		Colon cancer cell lines (*DLD-1* and *Caco-2*)	↓ Cell proliferation, cell cycle arrest at the G0/G1 phase
[25]		Osteosarcoma cell line (*MG-63*)	↓ Cell viability, induction of apoptosis after X-ray exposure, cell cycle arrest at the G2/M phase
[35]	Curcumin	Human colon cancer cells (*HCT116*)	↓ CDK2 kinase activity, ↓ cell proliferation, induction of cell cycle arrest at the G1 phase
[66]		Human pancreatic cancer cell line (BxPC-3)	↓ Activation of the EGF/ERK and EGF/AKT signalling pathways
[67]		Cervical cancer cell line *(HeLa*)	Anticancer activity
[36]	QCT	Human choriocarcinoma cells (*JAR* and *JEG3*)	↓ Cell proliferation, stimulation of ROS production, disruption of MMP^1^, cell cycle arrest at the sub-G1 phase
[37]		GC cells (SGC-7901 and *MGC-803*)	↓ viability of SGC-7901 and MGC-803 cells, induction of apoptosis, cell cycle arrest at the G2/M phase
[68]		GC cell (*AGS*, *MKN45*, *MKN7*, and *TMK1*)	Suppression of cell viability and reduction in tumour volume, ↓ concentration of GSH, MDA, and ROS
[39]		Retinoblastoma cells **(***Y79***)**	↓ Viability of cells, cell cycle arrest at the G1 phase, ↑ ROS
[69]		Oral cancer cell (*HSC-6* cells)	↓ Cell viability, tumour cell migration and invasive potential, ↓ concentration of MMP-2 and MMP-9
[70]		OSCC cells (*OSC20*, *SAS*, and *HN2*)	Suppression of cell viability, cell cycle arrest at the G2/M phase
[38]		KON oral cancer cells	Cell cycle arrest at the S and G2/M phase, activation of apoptosis, ↑ ROS
[71]		Prostate cancer cells (*LNCaP*, *PC3*, and *DU145*)	↓ Tumour cell growth (in a dose- and time-dependent manner), ↓ concentration of IL-1, IL-6, IL-8, and TNF-α
[42]	Resveratrol	Oral cancer cells (*H-357*) and human leukaemic monocyte cells (*THP-1*)	↓ Cytokine concentration in CSC-enriched cells; ↓ invasion, cell proliferation, and CSC expansion; ↓ metastatic markers (CD133, ALDH1, CXCR4) and angiogenic factors (MMP-2, iNOS, VEGF-A)
[45]		OSCC cells (*SCC-VII*, *SCC-25*, *YD-38*)	Cell cycle arrest at the G2/M phase; ↑ expression of Tyr15, cyclin A2, and cyclin B1 in cells
[72]		OSCC cells (*HSC-3*)	↓ Cell proliferation (cell cycle arrest at the G1 phase); ↓ expression of CDK4, CDK6, and cyclin D1; ↑ expression of p21^Cip1^ and p27^Kip1^
[45]		OSCC cells (*HSC-3*)	↓ Cell proliferation, induction of apoptosis (inhibition of the CBX7/AKT pathway and activation of p16 signalling cascades)
[73]		OSCC cells (*YD-9* and *YD-38*)	Inactivation of EGFR and downstream Zeb1 signalling
[74]		Human tongue squamous cell carcinoma cells (*CAL-27*, *SCC15*, and *SCC25*)	Inhibition of EMT-inducing transcription factors
[75]	LycT	Breast cancer cells (*MCF-7* and *MDA-MB-23*)	↑ Apoptosis
[52]		Human colon adenocarcinoma cells (*HT-29* and *LS174T*)	↓ Cell proliferation
[76]		Human colorectal cancer cells (*SW480*)	↓ mRNA TNF-α, IL-1β, IL-6, iNOS, COX-2; ↓ synthesis of NO and PGE_2_; ↓ NF-κB, IκB, and JNK protein expression (with ERK and p38 affected only at higher concentrations)
[51]		Human pancreatic cancer cell (*PANC-1*)	↓ ROS and MMP^1^, ↓ OCR, inhibition of NF-κB (reduced DNA binding, IκBα phosphorylation), ↓ NF-κB-dependent gene expression (cIAP1, cIAP2, survivin), ↑ caspase-3 activity and Bax/Bcl-2 ratio (induction of apoptosis)

Abbreviations: Nrf2, nuclear factor erythroid 2–related factor 2; TERT, human telomerase reverse transcriptase; TERC, human telomerase RNA component; CDK2, cyclin-dependent kinase 2; EGF, epidermal growth factor; ERK, extracellular signal-regulated kinase; AKT, protein kinase B; ROS, reactive oxygen species; MMP^1^, mitochondrial membrane potential; SGC-7901, stomach gastric cancer 7901; MGC-803, mucinous gastric cancer 803; GSH, glutathione; MDA, malondialdehyde; NF-κB, nuclear factor kappa B; MMP-2, metalloproteinase-2; MMP-9, metalloproteinase-9; IL-1, interleukin-1; IL-8, interleukin-8; TNF-α, tumour necrosis factor α; CSCs, cancer stem cells; CD133, prominin-1; ALDH1, aldehyde dehydrogenase 1; CXCR4, C-X-C chemokine receptor type 4; MMP, matrix metalloproteinase; iNOS, inducible nitric oxide synthase; VEGF-A, vascular endothelial growth factor A; Tyr 15, phosphorylated form of cyclin-dependent kinase 1; OSCC, oral squamous cell carcinoma; CDK4, cyclin-dependent kinase 4; CDK6, cyclin-dependent kinase 6; p21^Cip1^, cyclin-dependent kinase inhibitor 1; p27^Kip1^, cyclin-dependent kinase inhibitor 1B; CBX7, chromobox protein homolog 7; p16, cyclin-dependent kinase inhibitor 2A; EMT, epithelial–mesenchymal transition; EGFR, epidermal growth factor receptor; Zeb1, zinc finger E-box binding homeobox; mRNA, messenger RNA; IL-1β, interleukin-1β; IL-6, interleukin-6; COX-2, cyclooxygenase-2; NO, nitric oxide; PGE_2_, prostaglandin E2; NF-κB, nuclear factor-κB; IκB, inhibitor kappa B; JNK, c-Jun N-terminal kinase; OCR, oxygen consumption rate; IκBα; inhibitor of nuclear factor kappa-Bα; cIAP1; cellular inhibitor of apoptosis protein 1; cIAP2, cellular inhibitor of apoptosis protein 2; Bax, Bcl-2-associated X protein; Bcl-2, B-cell lymphoma 2; ↓, decrease; ↑, increase.

**Table 2:** A review of in vivo studies (animal models) on the effect of selected bioactive compounds on cancer cell proliferation and inflammatory processes.

Reference Source	Compound	Study Group/Tumour Type	Impact on the Organism/Mechanism of Action
[28]	BBR	*MKN-45* xenograft mice (GC)	↓ Tumour growth, ↓ concentration of IL-6
[84]		Mouse model of Lewis lung carcinoma cells	↓ Pulmonary metastasis
[85]		C57BL/6 mice (Lewis tumour xenograft mice)	↓ Tumour growth
[65]		*A549* tumour xenograft mice (lung cancer)	↓ Tumour growth
[23]		Female BALB/c nude mice (breast cancer)	↑ Antitumour efficacy of doxorubicin
[86]		C57BL/6J-*Apc*^Min/+^ mice	↓ Concentration of IL-6 and TNF-α in macrophages, ↓ EGFR-ERK pathway activity
[87]		Male BALB/c nude mice (liver tumour)	↓ Tumour growth
[88]	Curcumin	Female BALB/c mice (breast cancer)	↓ Cell migration, high antitumour efficacy
[89]		Female BALB/c mice (breast cancer)	↓ Tumour growth
[90]		Mice xenograft model (SW480) (colorectal cancer)	↓ Tumour growth, ↑ survival of mice, ↓ cell proliferation, induction of apoptosis, potential involvement of Wnt/β-catenin pathway inhibition
[91]		Female athymic (immunodeficient) nude mice (colorectal cancer) (*HCT116* and *HT-29*, *EF31* and *UBS109*)	↓ Tumour growth, enhancement of the therapeutic effects of oxaliplatin and 5-FU, ↓ expression of HIF-1α, COX-2, p-STAT-3, and VEGF
[92]		Rats (colorectal cancer and liver metastasis)	↓ Cell viability
[93]		Human colorectal cancer xenografts in nude mice (*SKOV3ip1*, *HeyA8*)	↓ Tumour growth by 49–55% when administered alone and by 77–96% in combination with docetaxel, ↓ proliferation, ↑ apoptosis, ↓ NF-κB, STAT3, and pro-angiogenic cytokines (VEGF, COX-2)
[29]	QCT	Female Sprague–Dawley rats (breast cancer)	Significant regression of hyperplastic lesions, ↑ apoptosis, ↑ expression of Bcl-2 protein, ↓ expression levels of Bax and p53
[94]		C57BL/6J female mice (breast cancer)	↓ STAT1 transcription factor expression in microglia No significant effect on breast tumour mass and volume under physiological conditions, ↓ tumour growth under stress conditions
[95]		TRAMP mice (prostate cancer)	↓ tumour volume and weight, ↓ concentration of Nrf2, modulation of genes involved in AR, PI3K/AKT, and PTEN signalling pathways, ↓ concentration of IGF1
[40]		Male Wistar rats (colon cancer)	↑ Concentration of SOD, CAT, GSH; ↑ expression of p53 and Bax proteins; ↓ concentration of Bcl-2
[96]		NOD/SCID mice (chronic lymphocytic leukaemia)	↓ Expression of Bcl-2, normalisation of miR-15a/16 levels, ↓ proliferation and migration of irradiated B-1 cells to the liver
[97]		Mice (human xenograft acute myeloid leukaemia models)	↓ Concentration of DNMT, ↓ expression of STAT3 and p-STAT3 proteins, ↓ concentration of HDAC1 and HDAC2, ↑ expression of DAPK1, Bax, APAF1, and BCL2L11
[41,98]		Mice (murine T-cell lymphoma)	↓ Cell viability, ↓ inflammation and angiogenesis through modulation of AKT signalling
[98]	LycT	Female Balb/c mice (hepatocellular carcinoma)	Stabilisation of metabolic and morphological parameters, ↑ numbers of apoptotic cells and macrophages
[99]		Female Balb/c mice (hepatocellular carcinoma)	Normalisation of haematological parameters; ↓ concentrations of TNF-α, IL-1β, and IL-6; enhancement of the antioxidant system
[100]		Laying hens: (ovarian cancer)	↓ Overall incidence of ovarian tumours, ↓ number and size of tumours, ↓ concentration of MDA
[101]		Rats (oesophageal cancer)	↑ Expression levels of PPARγ and caspase-3 proteins, ↓ expression of NF-κB and COX-2 protein in oesophageal tissue
[102]		Mice (Lewis lung carcinoma)	↓ Tumour size and weight, ↑ levels of IL-1 and IFN-γ, ↓ levels of IL-4 and IL-10 in spleen
[53]		Mice	Modulation of proliferation and apoptosis in keratinocytes exposed to UVB radiation
[54]		Male BALB/C mice (skin cancer)	↓ Cell proliferation, ↓ mRNA and protein expression of proliferating cell nuclear antigen
[42]	Resveratrol	Mice xenograft model (Balb/c mice) (oral cancer)	↓ Tumour size, ↓ expression of CD44 in liver, CXCR4 and Nanog in kidney and CXCR4 and VEGF-A in brain compared with control mice
[103]		Male Balb/c nude mice (cervical cancer)	↓ Tumour growth
[43]		Female athymic BALB/C nude mice (cervical cancer)	↓ Tumour size and weight, ↓ cell proliferation HeLa (in a dose-dependent manner)
[48]		Female athymic BALB/C nude mice (cervical cancer)	↓ Tumour size, ↓ expression of HPV E6 and E7, ↑ concentration of p53 and Rb1
[44]		Female athymic BALB/C nude mice (cervical cancer)	↓ Tumour size and weight, ↓ E6 and E7 transcription and translation, cell cycle arrest at the G1phase
[104]		Male Wistar rats (colorectal cancer)	Reduced body weight loss, ↓ concentration of ROS, ↑ concentration of GPx and GR
[95]		TRAMP mice (prostate cancer)	↓ Tumour size and weight; ↓ concentration of Nrf2; modulation of genes involved in AR, PI3K/AKT, and PTEN signalling pathways; ↓ concentration of IGF1

Abbreviations: IL-6, interleukin-6; TNF-α, tumour necrosis factor α; EGFR, epidermal growth factor receptor; ERK, extracellular signal-regulated kinase; 5-FU, 5-fluorouracil; HIF-1α, hypoxia-inducible factor 1α; COX-2, cyclooxygenase-2; p-STAT-3, phosphorylated signal transducer and activator of transcription-3; VEGF, vascular endothelial growth factor; NF-κB, nuclear factor-κB; STAT, signal transducer and activator of transcription-3; Bcl-2, B-cell lymphoma 2; Bax, Bcl-2-associated X protein; p53, tumour protein p53; STAT-1, signal transducer and activator of transcription-1; Nrf2, nuclear factor erythroid 2–related factor 2; AR, androgen receptor; PI3K/AKT, phosphoinositide 3-kinase/protein kinase B; PTEN, phosphatase and tensin homologue; IGF1, insulin-like growth factor; SOD, superoxide dismutase; CAT, catalase; GSH, glutathione; miRNA miR15a/16; DNMT, DNA methyltransferase; STAT-3, signal transducer and activator of transcription-3; HDAC1, histone deacetylase 1; HDAC2, histone deacetylase 2; DAPK1, death associated protein kinase 1; APAF1, apoptotic peptidase activating factor 1; BCL2L11, Bcl-2-like protein 11; AKT, protein kinase B; IL-1β, interleukin-1β; PPARγ, peroxisome proliferator-activated receptor γ; IL-1, interleukin 1; IFN-γ, interferon γ; IL-4, interleukin 4; IL-10, interleukin 10; CD44, cluster of differentiation 44; CXCR4, C-X-C motif chemokine receptor 4; VEGF-A, vascular endothelial growth factor A; HPV E6, human papillomavirus early protein 6; HPV E7, human papillomavirus early protein 7; Rb1, retinoblastoma protein 1; ROS, reactive oxygen species; 8-okso-dG, 8-hydroxy-2′-deoxyguanosine; AP, apurinic/apyrimidinic; MDA, malondialdehyde; OGG1, 8-oxoguanine DNA glycosylase; APE1, apurinic/apyrimidinic endonuclease 1; XRCC1, X-ray repair cross-complementing protein 1; GPx, glutathione peroxidase; GR, glutathione reductase; GST, glutathione s-transferase; ↓, decreased; ↑, increased.

**Table 3:** A review of in vivo studies (involving humans) on the effect of selected bioactive compounds on cancer cell proliferation and inflammatory processes.

Reference Source	Compound	Study Group/Tumour Type	Impact on the Organism/Mechanism of Action
[112]	Curcumin	Patients with advanced metastatic breast cancer	51% Objective response rate, ↓ fatigue, ↑ patients’ self-assessed overall physical performance
[30]		Patients with breast cancer	Cell cycle arrest, induction of apoptosis (induction of the p53/p21 pathway)
[113]		Participants with clinical symptoms of oral leukoplakia	A clinical response (complete or partial) was observed in 67.5% of the study participants undergoing chemopreventive treatment for oral leukoplakia after 6 months of intervention
[114]		Participants with pancreatic cancer	↑ Effectiveness of GEM treatment
[115]		Participants with PCa	High response rates during supportive treatment, with good patient acceptability and tolerability
[116]		Smokers with eight or more ACF	40% Reduction in the number of ACF following a 4 g dose
[117]		Patients with familial adenomatous polyposis	No significant changes in the number or size of polyps
[118]	BBR	Patients with chronic lymphocytic leukaemia	↓ Cell activation, ↓ anti-apoptotic protein expression (Mcl-1, Bcl-XL), disruption of oxidative phosphorylation, ↑ cellular sensitivity to venetoclax
[119]		Patients with B-chronic lymphocytic leukaemia	↓ Bcl-2 levels and ROR1 receptor expression, ↑ Bax/Bcl-2 ratio
[120]		Patients with adenomyosis	↓ Cell proliferation (in a dose- and time-dependent manner), cell cycle arrest at the G0/G1 phase, ↑ apoptosis, ↓ expression of IL-6, IL-8, TGF-β, EGF, VEGF, and MMP-2
[121]	LycT	Men with PCa	↓ PSA, improvement in quality of life, inhibition of tumour progression
[122]		Patients with non-metastatic PCa	↓ Median PSA levels
[123]		Participants with head and neck cancer	Hypomethylation of CD8A and ↑ CD8 and CD8+ T cells, hypermethylation of CD40LG and TEC
[124]	QCT	Patients with lung cancer	Regulation of miRNA (let-7, miR-146a), ↓ miR-17 (oncogenic)
[125]	Resveratrol	Patients with colorectal cancer	↓ Proliferation (Ki67 ↓), high tissue concentration in the intestinal epithelium

Abbreviations: p53, tumour protein p53; p21, cyclin-dependent kinase inhibitor 1A; GEM, gemcitabine; ACF, aberrant crypt foci; Mcl-1, myeloid cell leukaemia 1; Bcl-XL, B-cell lymphoma-extra large; Bcl-2, B-cell lymphoma 2; Bax, Bcl-2-associated X protein; ROR1, receptor tyrosine kinase; IL-6, interleukin-6; IL-8, interleukin-8; TGF-β, transforming growth factor-β; EGF, epidermal growth factor; VEGF, vascular endothelial growth factor; MMP-2, metalloproteinase-2; PSA, prostate-specific antigen;, CD40LG, hypermethylation of CD40 ligand; TEC, Tec protein tyrosine kinase; miRNA, microRNA-17; PCa, prostate cancer; ↓, decreased; ↑, increased.
